# Conservation agriculture has no significant impact on sheep digestive parasitism

**DOI:** 10.3389/fvets.2023.1244355

**Published:** 2023-09-21

**Authors:** Sihem ElHamdi, Limam Sassi, Mourad Rekik, Mokhtar Dhehibi, Hatem Cheikh M'hamed, Mohamed Gharbi

**Affiliations:** ^1^Ruminant Internal Medicine Service, Institution de la Recherche et de l'Enseignement Supérieur Agricoles, École Nationale de Médecine Vétérinaire de Sidi Thabet, Université de la Manouba, Sidi Thabet, Tunisia; ^2^Laboratoire de Parasitologie, Institution de la Recherche et de l'Enseignement Supérieur Agricoles, École Nationale de Médecine Vétérinaire de Sidi Thabet, Université de la Manouba, Sidi Thabet, Tunisia; ^3^International Center for Agricultural Research in the Dry Areas (ICARDA), Amman, Jordan; ^4^Institut National des Recherches Agronomiques de Tunis (INRAT), Tunis, Tunisia

**Keywords:** conservation agriculture, conventional agriculture, digestive parasites, sheep, Tunisia

## Abstract

Conservation agriculture (CONS A) is a sustainable agriculture system based on crop rotation with no tillage. It has various environmental advantages compared to conventional agriculture (CONV A): decreased water evaporation, erosion, and CO_2_ emissions. In this first study of its kind, we aim to evaluate the impact of this type of agriculture on sheep gastrointestinal parasites. Two lamb groups aged between 5 and 10 months were randomly included to graze separately on CONS A and CONV A pastures. Each group was composed of two batches of three lambs, and these were followed up for two rearing months. Liveweight, hematological parameter variation, and digestive parasites were studied. At the end of the study period, lambs were slaughtered the carcass yield was determined, and a helminthological autopsy was performed on the digestive tracts of the animals to estimate different parasitological indicators. There was no difference between lambs reared on CONS A and those reared on CONV A for all parasite indicators (infestation intensity, abundance, and prevalence). The same trend was also obtained for hematological parameters, liveweight evolution, and carcass yield. These results prove that there is no impact of CONS A on the sheep's digestive parasitism. Further studies are needed to support these findings on larger animal samples and to investigate the impact of conservation agriculture on other parasite species. Similar studies could also be conducted on ruminant species.

## 1. Introduction

Conservation agriculture (CONS A) or regenerative agriculture is a sustainable model that does not disturb the ecosystem and preserves natural resources (1). It contributes to the preservation of the physico-biological properties of the soil and its mesofauna and microfauna, which has a positive impact on soil fertility and productivity.

Owing to the presence of a permanent vegetative cover, CONS A protects the soil from erosion and reduces the evaporation of water. However, it decreases the release of CO_2_ gases from the ground (2), reducing climate warming (3–7).

The livestock-crop association leads to several ecological benefits. It replaces chemical fertilizers with organic fertilizer made from livestock droppings that are naturally rich in minerals (phosphorus and nitrogen). This generates a balance or a mutual profile between crops and livestock populations (8). Besides, it was proven that there is an efficiency of crop-livestock production systems under CONS A with the guarantee of sustainable food security in Tunisian dry areas (9, 10).

Numerous studies have proven that parasitic diseases are widespread throughout the world. They cause high financial losses that were estimated at ~1.8 billion euros in 18 countries of the European Union (11). In Tunisia, parasites represent an important health problem in sheep since the parasitic fauna is very diversified, with a high infestation/infection prevalence (a large proportion of animals have parasites) and intensity (high parasitic burdens in the majority of animals).

Moreover, sheep digestive parasitism in CONV A has a high impact due to the high infestation prevalence (a high proportion of animals are infected by these parasites), the high intensity (infected animals have high parasitic burdens), and the parasite diversity (a very large fauna of parasites are infecting animals). For example, Tariq et al. (12) mentioned that more than 67% of sheep were infected by gastrointestinal nematodes (GIN) in CONV A of the Kashmir Valley, India, and Krishnamoorthy et al. (13) reported an overall prevalence of 65% in CONV A grazing sheep in semiarid areas of India between 1998 and 2021. Waruru et al. (14) reported a high prevalence of GIN (51.6%), *Eimeria* spp. (31.5%), and tapeworms (28%) in Machakos District, Kenya.

Indeed, a very high infection prevalence by gastrointestinal nematodes was reported in Tunisia. They affect between 70 and 100% of tested animals (15, 16). Animals infected by gastrointestinal nematodes shed a very high number of eggs that hatch and give three successive larval instars daily in their feces. The probability that these four stages survive in the environment closely depends on three abiotic factors (temperature, hygrometry, and oxygenation). For this reason, soil management dramatically influences the epidemiological pattern of digestive gastrointestinal parasites, including protozoa (mainly *Eimeria* spp.), intestinal imaginal cestodes (adult tapeworms), and gastrointestinal nematodes.

The aim of the present comparative preliminary study was to investigate the impact of CONS A on these three groups of sheep digestive parasites, the hematological indicators of anemia, and weight indicators (liveweight and yield carcass). These outputs will provide animal decision-makers with a very important dataset about the potential parasitic management that could be recommended when sheep graze on CONS A.

## 2. Materials and methods

### 2.1. Study farm

**Figure 1:**
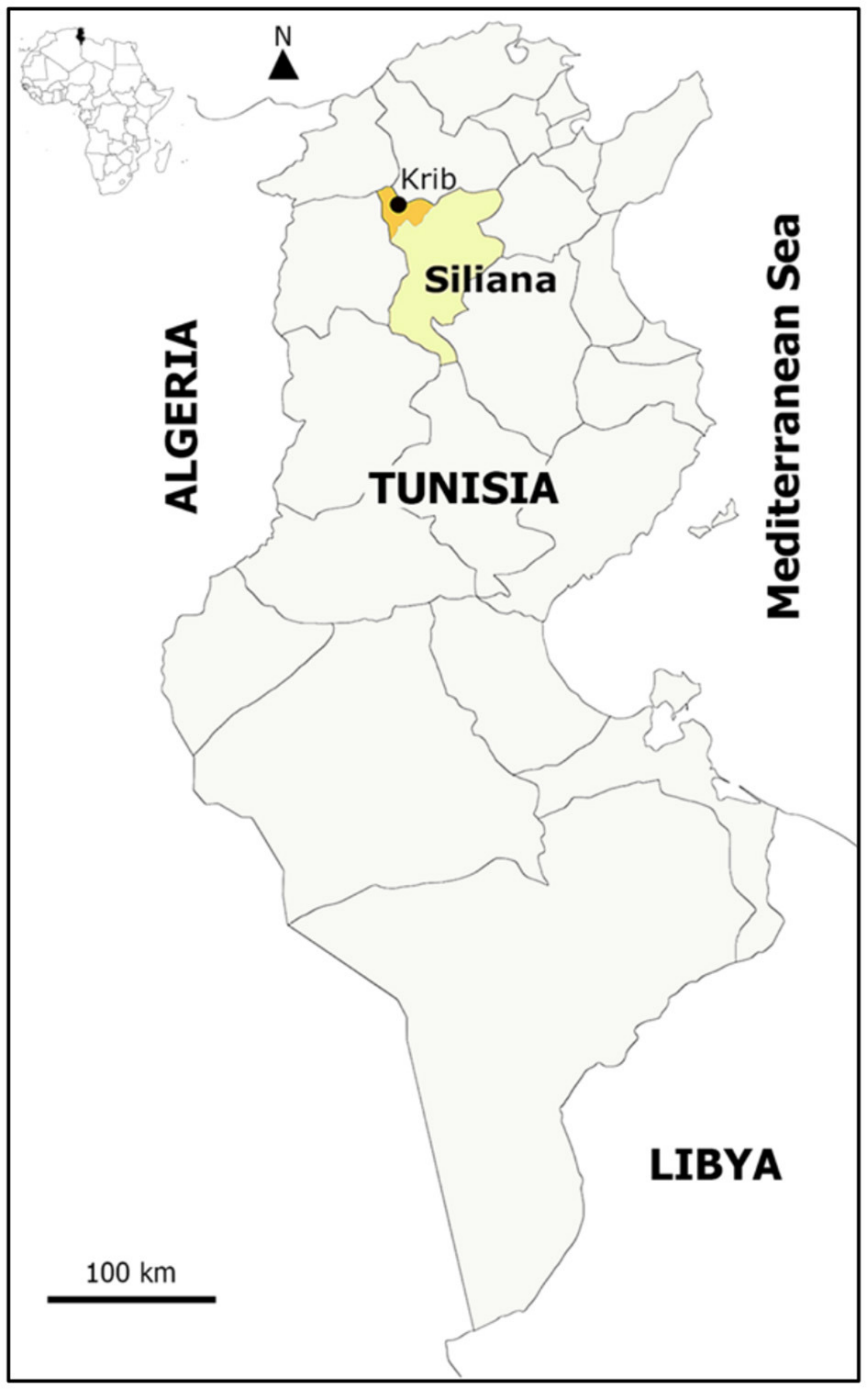
Geographical location of the study farm in Siliana governorate, North Tunisia.

The present study was conducted on a private farm in the Krib locality, Siliana district, Northwest Tunisia (Latitude: 36.374471 E; Longitude: 9.175250 N) (Figure 1).

**Figure 2:**
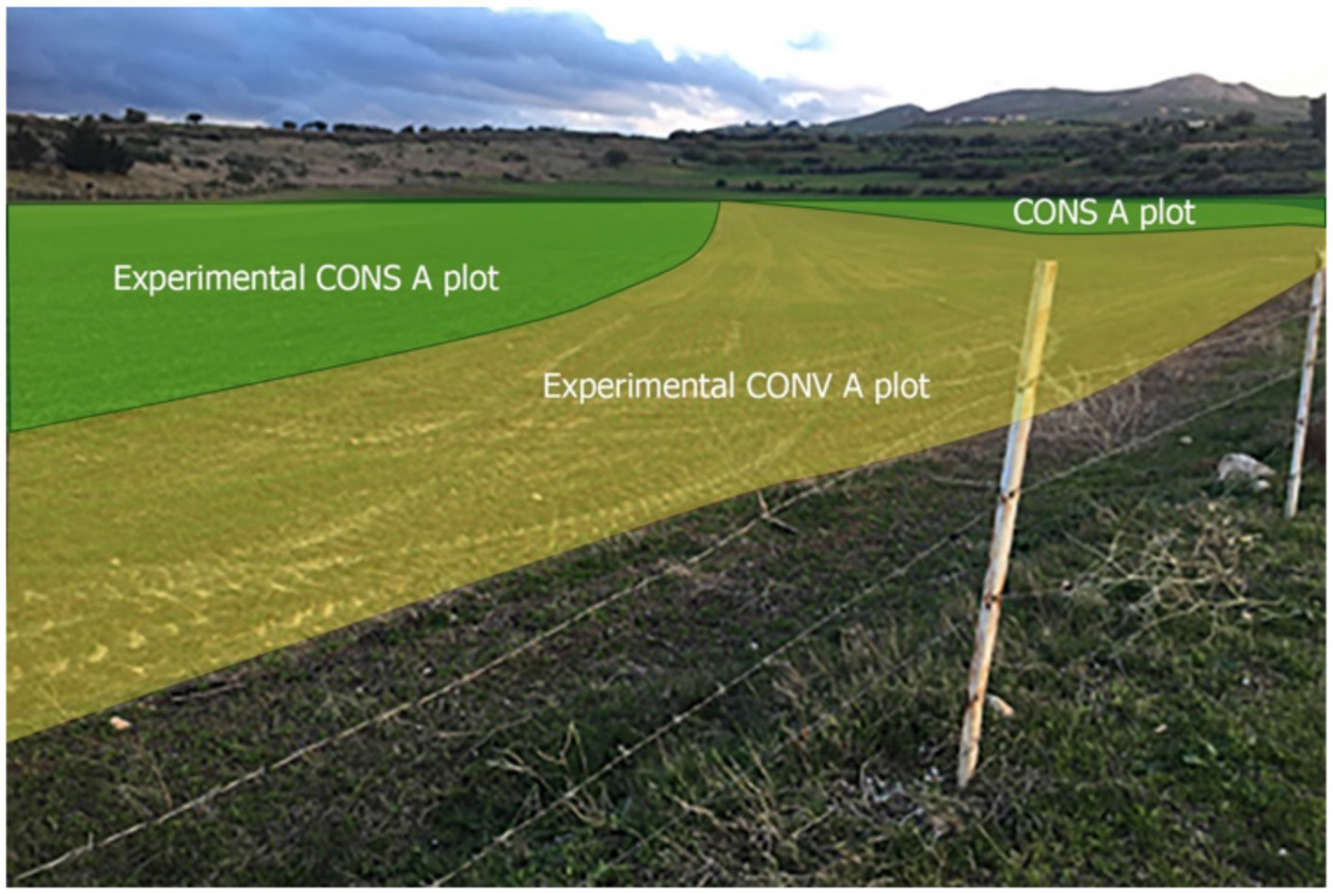
Landscape of CONS A and CONV A plots grazed by experimental lambs in the present study in January.

The Krib locality has a Köppen BSk climate type with an average annual rainfall between 250 and 600 mm and mean winter and summer temperatures of 17.8 and 35°C, respectively (17). The study land consists of two contiguous plots, one used for conservation agriculture (CONS A) and the other for conventional agriculture (CONV A) (Figure 2). Agricultural activities were similar and performed at the same time in both plots. Both of them were planted with oats (*Avena sativa*), vetch (*Vicia sativa*), sulla (*Hedysarum coronarium*), and alfalfa (*Medicago sativa*).

### 2.2. Animals

**Figure 3:**
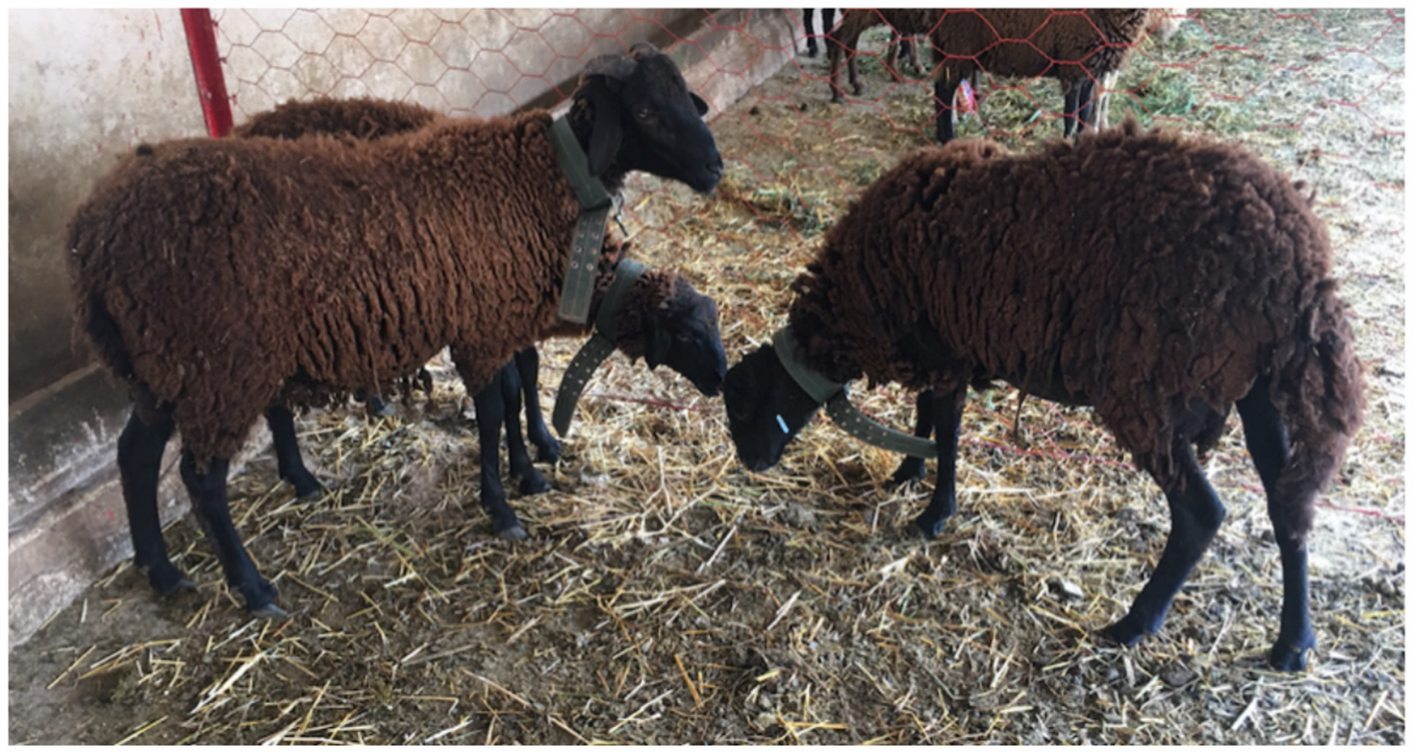
Noire de Thibar experimental lambs in their boxes.

Two batches, each containing six male lambs, were randomly selected from a herd of sheep consisting of 130 Noire de Thibar, Queue Fine de l'Ouest, and cross-breeds. Lambs between 5 and 10 months with a mean liveweight of 24 kg (range: 16–32 kg) were sampled for this study. Animals were vaccinated against enterotoxaemia (Ovipan F^®^, MCI Santé Animale, Morocco) (two subcutaneous injections of 2 ml/animal at 1-month interval). They were also treated with 7 mg/kg albendazole (Dalben^®^ 1.9, CEVA, France) in late January 2021. Each batch of lambs was randomly divided into two groups, each comprising three male lambs, and maintained in two separate boxes (Figure 3). After a month-long adaptation period, the two groups were randomly placed on pastures for 2 months, one on a conservation agriculture (CONS A) plot and the other on a conventional agriculture (CONV A) plot. Both batches of lamb grazed daily in fenced 25 m^2^ plots for 6 to 7 h for 3 days, except during rainy days, when they were kept in their boxes. As a food supplement, each lamb received *ad libitum* oat vetch hay and ~200 g of concentrate.

### 2.3. Sampling

Every 2 weeks, lambs were clinically examined, weighed, and sampled (5 ml of blood in EDTA tubes and at least 10 g of feces).

Red blood cell count (RBC) (10^9^/mL), haematocrit (Ht) (%), and hemoglobin (Hb) (g/dL) were estimated using an auto-hematology analyzer BC-2800Vet^®^ (Shenzen Mindray Bio-Medical Electronics Co., Ltd., China).

All fecal samples were examined qualitatively (using the flotation technique) and quantitatively (using the McMaster technique) for the presence of gastrointestinal parasites. The latter allowed the estimation of infection intensity that was expressed as eggs per gram (epg) of gastrointestinal nematodes, coccidian oocysts, and whipworms (*Trichuris ovis*) (18).

After 2 months of grazing, the lambs were slaughtered. Gastrointestinal tracts, lungs, livers, and epiploons were collected, and each carcass was weighed. The collected organs were thoroughly examined and dissected for the presence of lesions. Each portion of the gastrointestinal tract was separated and longitudinally opened. The digestive mucosa was thoroughly washed, and all the content was collected in a bucket. All cestodes and nematodes were collected and washed in 70% ethanol. All the collected parasites were conserved in identified tubes containing 70% ethanol and then stored at +4°C until studied. According to taxonomic criteria, nematodes and segments of adult cestodes were counted and identified (19).

### 2.4. Parasitological indicators

The following parasitological indicators were estimated (20):

Total Worm Count (TWC) = total number of nematode species found in one examined gastrointestinal tract. A natural logarithmic transformation [Ln (n + 1)] was used in the figures.

Infestation prevalence (%) = 100 × number of infested lambs/number of examined lambs.

Infestation intensity = the number of parasites in the gastrointestinal tract/number of infested lambs.

Infestation abundance = the number of parasites in the gastrointestinal tract/number of examined lambs.

### 2.5. Statistical analyses

During visits, the mean relative variation was used to compare the variation of lambs' weight, haematocrit, hemoglobin, and blood cell count. The relative variation was estimated as follows:

Mean relative variation (%) = 100 × [value at visit (n + 1) - value at visit (n)]/[value at visit (n)].

Since the number of lambs was low, the comparison of the prevalence of tapeworm infestation during visits between the two lamb batches was performed with Fisher's exact test. The infestation intensity and abundance (EPG, OPG, and whipworms) of the two batches of lambs during the five visits were determined using the Wilcoxon-Mann-Whitney test.

The Kruskal–Wallis test was used to compare the infestation intensity of lambs within the same group from the first to the fifth visit to compare the intra-group infestation intensity.

All tests were considered significant at a 5% threshold (21, 22).

## 3. Results

### 3.1. Relative variation of lambs' liveweights

**Figure 4:**
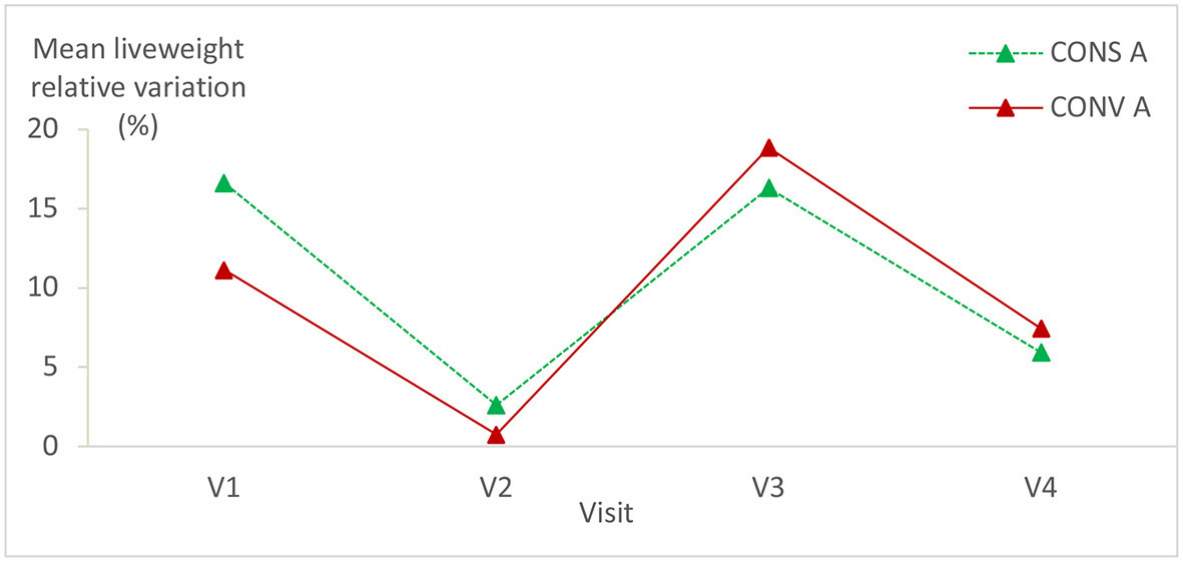
Mean relative variation (in %) of lambs' liveweights in CONS A and CONV A. CONS A, Conservation Agriculture; CONV A, Conventional Agriculture.

The mean relative variation of lambs' liveweight had exactly the same trend in both animal groups. It decreased considerably on the second visit. There was no statistically significant difference between the liveweights in the two animal groups (Table 1; Figure 4). It is worth mentioning that there is a significant statistical difference in liveweight relative variation in each batch during all visits (Table 1) (*P* = 0.01 for both lambs' batches).

**Table 1:** *P*-values of lambs' mean weight relative variation, hematological parameters relative variation, and McMaster technique between the two batches of lambs.

	**CONS A vs. CONV A**					**CONS A visit 1 vs. visit 5**	**CONV A visit 1 vs. visit 5**
	**Visits**						
**Parameter**	**Visit 1**	**Visit 2**	**Visit 3**	**Visit 4**	**Visit 5**		
Relative variation of lambs' weight	NA	0.937	0.589	0.589	0.818	**0.01**	**0.01**
**Relative variation of hematological parameters**							
Hemoglobin (Hb)	NA	0.818	0.937	0.394	0.589	**0.04**	0.22
Haematocrit (Ht)	NA	0.132	0.818	0.485	0.485	**0.02**	0.64
Red blood cells count (RBC)	NA	0.24	0.818	0.669	0.589	0.12	0.98
**McMaster technique**							
Oocyst per gram (Opg)	0.24	0.792	0.589	0.093	1	**0.01**	0.49
Tapeworms	0.72	0.61	0.5	0.73	0.73	0.99	0.93
Whipworms	0.065	0.662	0.818	0.394	0.18	0.74	0.54
Egg per gram strongyles except for whipworms (epg)	0.589	0.662	0.937	0.18	1	1	0.14

In bolded characters are statistically significant values. NA, Not Applicable.

The carcass yield was low for lambs in both types of agriculture (44.5 and 45.3% for CONS A and CONV A, respectively) and almost did not exceed the lower limit of the range of carcass yield in sheep (between 45 and 60%) (23). Moreover, there was no statistically significant variation in carcass yield between the two lamb batches (*P* = 0.39).

### 3.2. Relative variation of hematological parameters

**Figure 5:**
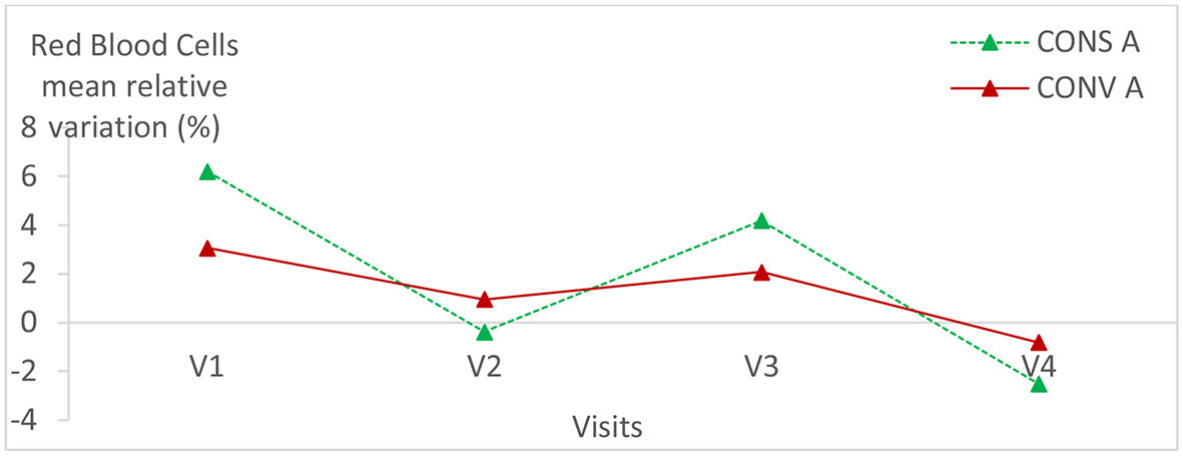
Mean relative variation of red blood cells in the two lamb groups according to visits. CONS A, Conservation Agriculture; CONV A, Conventional Agriculture.

**Figure 6:**
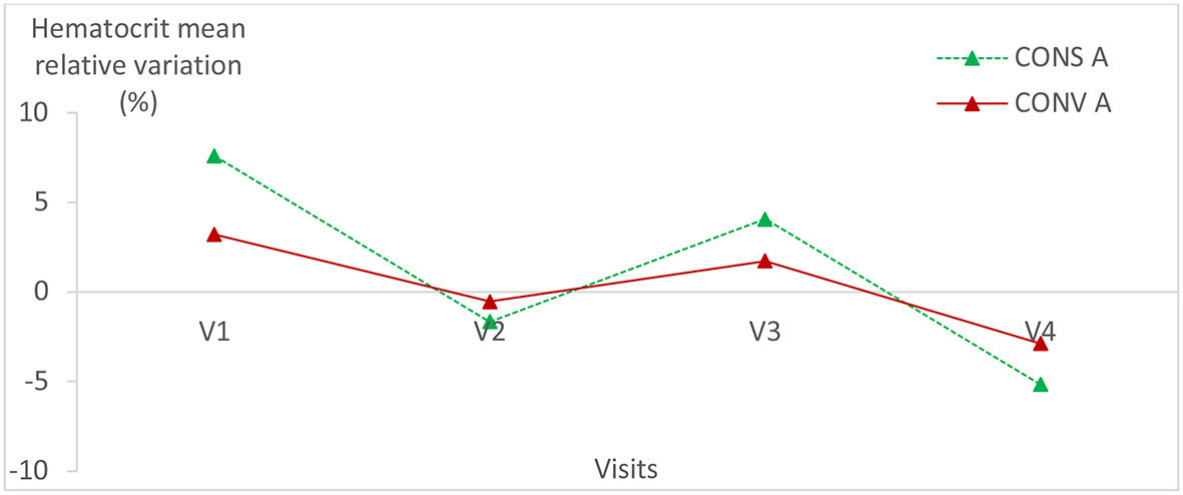
Mean relative variation of haematocrit in the two lamb groups according to visits. CONS A, Conservation Agriculture; CONV A, Conventional Agriculture.

**Figure 7:**
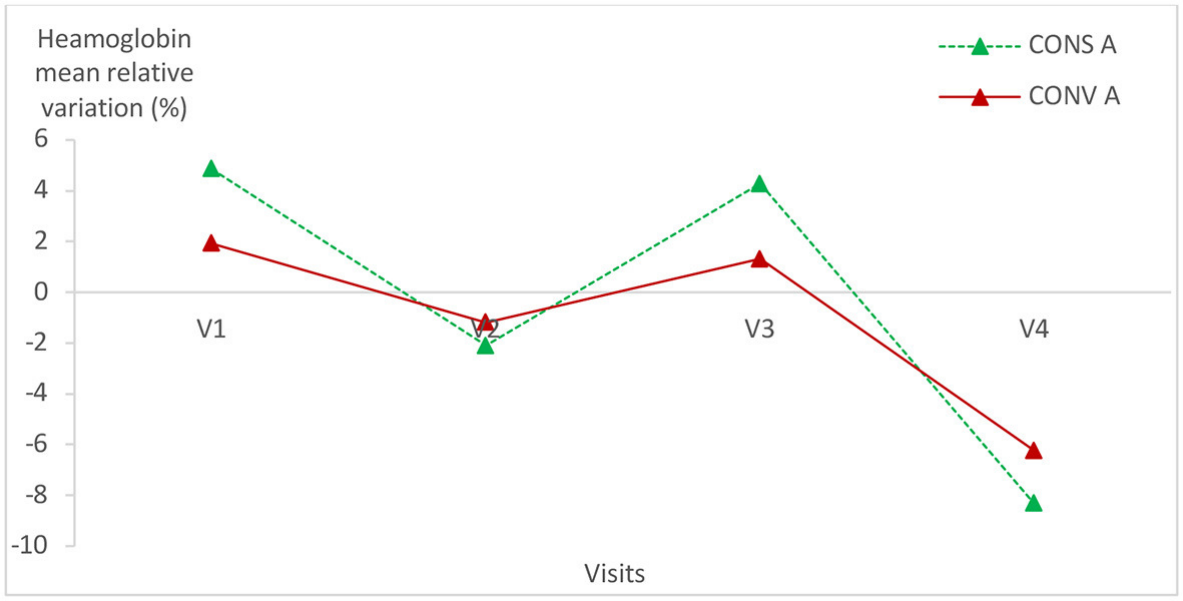
Mean relative variation of hemoglobin in the two lamb groups according to visits.

Hematological parameters were within the normal values of lamb blood parameters in all animals of both groups (24, 25). The hematological parameters had the same variation trend in the two lamb groups (Figures 5–7; Table 1) (*p* > 0.05).

There was no statistically significant difference within each group of lambs except for the hemoglobin relative variation in lambs kept in CONS A (*P* = 0.04) (Table 1). It increased from the first to the fourth visit and then decreased during the last visit.

### 3.3. Coproscopic results

**Figure 8:**
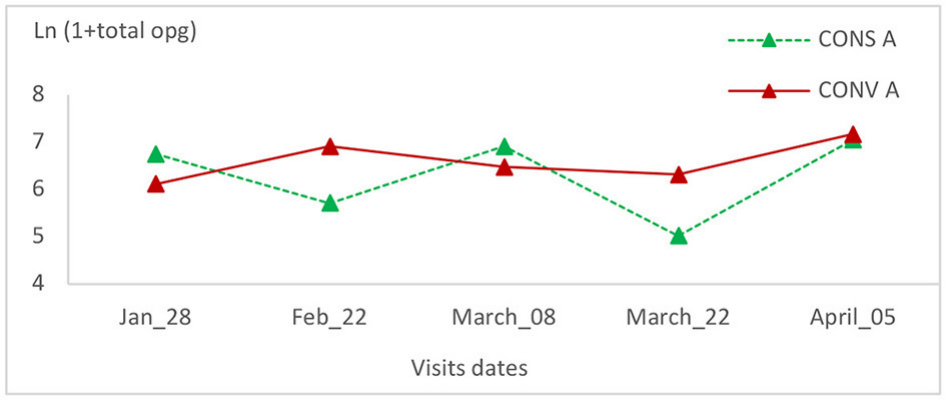
Total oocyst count intensity variation in the two lamb groups in the first batch.

The total coccidia oocyst count showed no significant change in the CONV A lamb group of the first batch (Figure 8). In the CONS A group, this value decreased on the second and fourth visits (Figure 8). The total oocyst counts within this group showed a significant statistical variation (*P* = 0.01) (Table 1).

**Figure 9:**
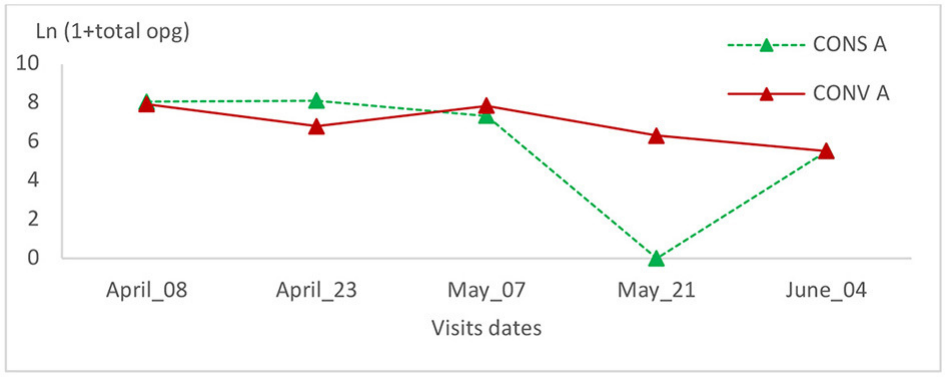
Total oocyst count intensity variation in the two lamb groups in the second batch.

The prevalence rate of tapeworms (*Moniezia* spp.) did not change during all visits, and no statistically significant variation was observed (*p* > 0.99) (Table 2). In another side, the total oocysts count of the CONV A lambs' group, showed the same trend and no statistical significant variation has been recorded (Figure 9).

**Table 2:** Prevalence rate of digestive parasites in CONS A and CONV A lambs per visit.

	**Visit 1**			**Visit 2**			**Visit 3**			**Visit 4**			**Visit 5**		
**Coprological parameters**	**CONS A**	**CONV A**	***P*** **-value**	**CONS A**	**CONV A**	***P-*** **value**	**CONS A**	**CONV A**	***P*** **-value**	**CONS A**	**CONV A**	***P-*** **value**	**CONS A**	**CONV A**	***P-*** **value**
Egg per gram (Epg)	2/6 (33.33 ± 0.19)	2/6 (33.33 ± 0.19)	> 0.99	1/6 (16.67 ± 0.15)	2/6 (33.33 ± 0.19)	> 0.99	1/6 (16.67 ± 0.15)	2/6 (16.67 ± 0.15)	> 0.99	1/6 (16.67 ± 0.15)	4/6 (66.67 ± 0.19)	0.24	1/6 (16.67 ± 0.15)	1/6 (16.67 ± 0.15)	> 0.99
Oocysts per gram (Opg)	6/6 (100 ± 0)	6/6 (100 ± 0)	NA	5/6 (83.33 ± 0.15)	6/6 (100 ± 0)	NA	6/6 (100 ± 0)	6/6 (100 ± 0)	NA	2/6 (33.33 ± 0.19)	5/6 (83.33 ± 0.15)	0.24	5/6 (83.33 ± 0.15)	4/6 (66.67 ± 0.19)	> 0.99
Whipworm eggs (*Trichuris ovis*)	2/6 (33.33 ± 0.19)	0/6 (0 ± 0)	NA	2/6 (33.33 ± 0.19)	1/6 (16.67 ± 0.15)	> 0.99	2/6 (33.33 ± 0.19)	1/6 (16.67 ± 0.15)	> 0.99	2/6 (33.33 ± 0.19)	0/6 (0 ± 0)	NA	3/6 (50 ± 0.2)	0/6 (0 ± 0)	NA
Tapeworm eggs (*Moniezia sp*.)	2/6 (33.33 ± 0.19)	2/6 (33.33 ± 0.19)	> 0.99	2/6 (33.33 ± 0.19)	3/6 (50 ± 0.2)	> 0.99	2/6 (33.33 ± 0.19)	3/6 (50 ± 0.2)	>0.99	2/6 (33.33 ± 0.19)	2/6 (33.33 ± 0.19)	> 0.99	2/6 (33.33 ± 0.19)	2/6 (33.33 ± 0.19)	> 0.99

CONS A, Conservation Agriculture; CONV A, Conventional Agriculture; NA, Not Applicable; SE, Standard Error.

**Figure 10:**
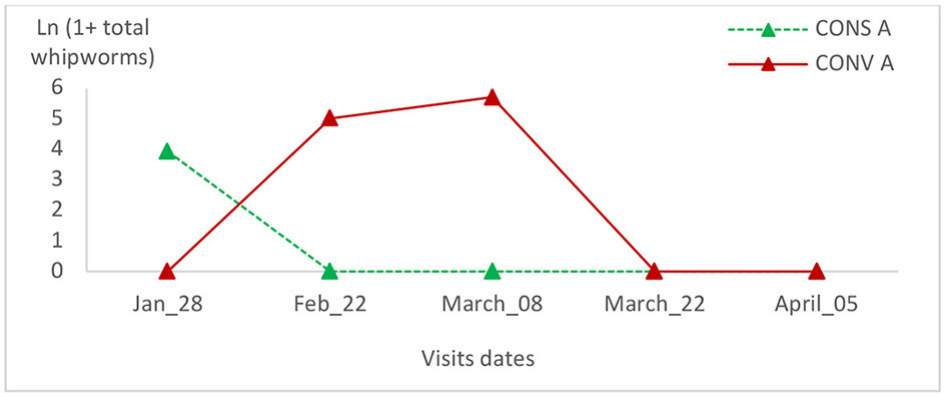
Total whipworm intensity variation in the two lamb groups in the first batch.

**Figure 11:**
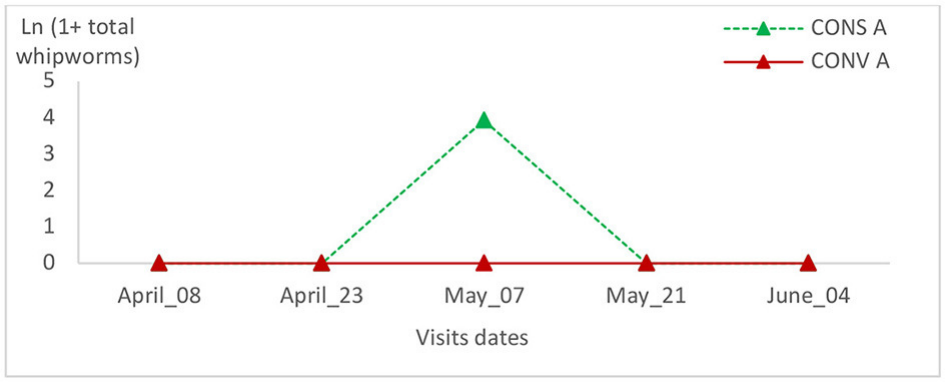
Total whipworm intensity variation in the two lamb groups in the second batch.

The eggs' whipworms relative variation in the CONV A lamb group of the first batch peaked during the third visit and reached zero at the last visit (Figure 10). This value was zero from the second visit in the CONS A lamb group (Figure 10). In the second batch, the eggs' whipworms' relative variation was zero throughout the visits in the CONV A lamb group (Figure 11). There were no statistically significant differences between lambs in the two groups during all visits or in the same batch (Table 2).

**Figure 12:**
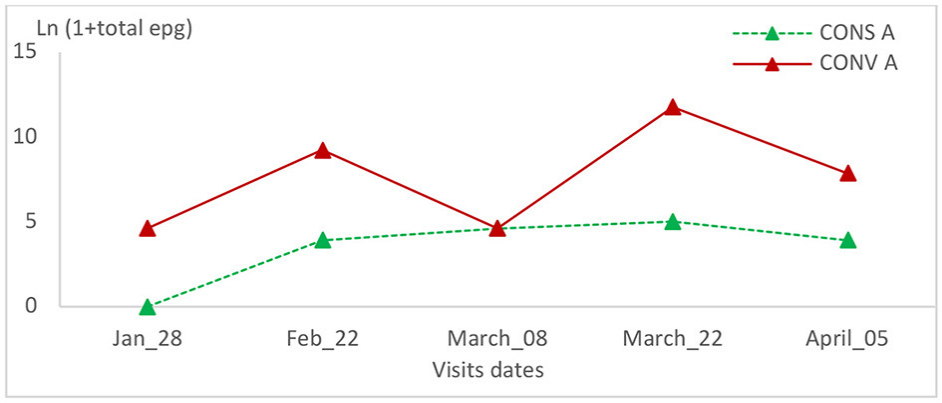
Total epg intensity variation in the two groups of lambs in the first batch.

**Figure 13:**
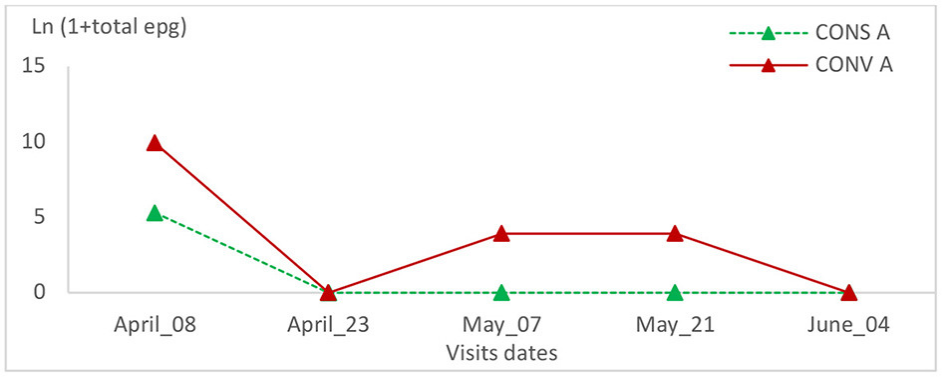
Total epg intensity variation in the two groups of lambs in the second batch.

The relative variation of epg of the other gastrointestinal nematodes was not statistically significant between the two lamb groups or within the same group during all visits (Tables 1, 2 and Figures 12, 13).

The probability of observed infestation prevalence in CONV A and CONS A was 50 ± 0.09% (extreme values: 35.5 and 71.1%) and 56.67 ± 0.09% (extreme values: 39.6 and 73.8%), respectively.

### 3.4. Helminthologic necropsy

A total of 905 helminth specimens were collected from the 12 slaughtered lambs. Among these helminths, abomasum nematodes were predominant, mainly *Ostertagia sp.*, which were collected from all the lambs of both groups (Figure 13). It represented 94.25% of the total number of parasites (853 worms). The total worm count (TWC) varied between 9 and 190 worms per lamb.

There was no statistical difference between the lamb groups for infestation intensity and abundance (Table 3).

**Table 3:** Infestation prevalence, intensity, and the abundance of different gastrointestinal parasites in the two lamb groups.

**Parasites**	**Infestation prevalence (%** ± **SE)**			**Infestation intensity**			**Infestation abundance**		
	**CONS A**	**CONV A**	***P-*** **value**	**CONS A**	**CONV A**	***P-*** **value**	**CONS A**	**CONV A**	***P-*** **value**
*Ostertagia sp*.	100 ± 0	100 ± 0	NA	65.16	77	0.873	65.16	77	1
*Marshallagia marshalli*	16.67 ± 0.15	0 ± 0	>0.05	1	NA	NA	0.16	0	0.699
*Nematodirus*	16.67 ± 0.15	16.67 ± 0.15	>0.05	3	1	NA	0.5	0.16	0.937
*Cooperia*	33.33 ± 0.19	0 ± 0	>0.05	8.5	NA	NA	2.83	0	0.394
*Trichuris ovis*	50 ± 0.2	16.67 ± 0.15	>0.05	4.66	1	NA	2.33	0.16	0.24
*Chabertia ovina*	16.67 ± 0.15	0 ± 0	>0.05	14	NA	NA	2.33	0	0.699
*Skrjabinema ovis*	0 ± 0	16.67 ± 0.15	>0.05	NA	1	NA	0	0.16	0.699
*Moniezia sp*.	33.33 ± 0.19	33.33 ± 0.19	>0.05	7	15.5	NA	2.33	5.16	0.818

NA, Not Applicable.

## 4. Discussion

The environmental benefits of CONS A, especially regarding climate change and land preservation, are very high, and governments should encourage this type of agriculture to maintain the sustainability of agricultural activity and reduce its negative impact on the environment (6, 26, 27). As part of combining crops with livestock, particularly in semiarid areas, CONS A and sheep farming can be harmoniously associated (10, 28).

Parasitic infections constitute a burden in extensive small ruminant breeding because of the subsequent considerable economic losses (11). Thus, parasitic infections are widespread, infestation rates are mostly high (15, 16), and sheep productivity is widely constrained (29).

This low productivity is due to negative repercussions on the general metabolism of infested animals. These effects include decreased appetite, slowed and disrupted nutrient digestion, reduced growth rate, and, in advanced stages, disturbed hematological parameters (30).

On the other side, unless CONS A is a sustainable farming system that has ecological benefits and fits perfectly with the breeding of small ruminants, it risks changing the resistance of certain parasites in the external environment compared to that in CONV A and may alter the epidemiology of parasitic diseases.

Everything mentioned above prompted us to undertake this study. Thus, our study aims to follow up and compare the hematological parameters, the relative variation in live weight, and the gastrointestinal parasite infestation parameters in two batches of lambs grazing in CONS A and CONV A, respectively, during two grazing cycles to determine whether this hypothesis is valid.

To the best of our knowledge, to date, this is the first study to investigate the effect of CONS A on sheep digestive parasitism. We found that the mean relative variation of lambs' liveweight decreased in the second visit in the two animal groups. This could be attributed to the modification of the food regime during the adaptation period. The absence of a statistical difference between lambs' liveweights in the two batches indicates that CONS A did not negatively impact the parasitism or the growth rate of sheep. A statistically significant variation in liveweights was observed in both animal groups during the five visits. This is attributed to the increase in liveweight following the maintenance of lambs' growth, even between the ages of 6 and 12 months (31).

Moreover, the mean carcass yield of the CONS A lamb group (445 g/kg) was slightly lower than the normal yield values for fattening lambs (between 450 and 600 g/kg of body weight) (23). This variation was probably due to genetic traits, gender, age, birth weight, and feeding level (28). There was no statistically significant difference in the mean carcass yield between the two animal batches (CONS A and CONV A lamb batches). These results indicate that growth is not affected when lambs are kept in CONS A pastures. Further studies involving larger lamb samples are needed to consolidate this finding.

No statistically significant difference was found in hematological parameters between the two lamb batches. This proves that the digestive parasite infection level had no impact on the hematological parameters either in CONS A or in CONV A and, consequently, on the metabolism of the studied lambs.

No statistically significant difference was found between the two lamb groups concerning infestation prevalence, intensity, or the abundance of all parasites found. The parasitological study did not show any statistically significant difference regarding infection by *Eimeria*, whipworms (*T. ovis*), digestive strongyle eggs, or tapeworms in the two lamb batches. These results confirm that pasturing on CONS A crops has no negative impact on lambs' digestive parasitism.

*Eimeria* fecal elimination during the grazing period showed the same trend in both types of agriculture, with a higher infection intensity during the wet period. These results are in agreement with those found in grazing sheep in semiarid areas in Brazil (32). There was a statistically significant variation in total oocyst count in lambs grazing on CONS A pasture. The progressive decrease in the total oocyst count in the two batches of CONS A lambs' infection intensity could be explained by a progressive installation of specific anti-*Eimeria* immunity. This trend was due to the separation of experimental lambs from adult sheep, leading to the absence of lamb contamination from carrier adult sheep (33). The relative increase in total oocyst count at the last visit could be explained by an increase in ambient humidity and temperature during the spring.

The prevalence rate of worms in the two lamb batches showed the same trend. It varied between 16.7 and 66.7% in CONS A and CONV A lamb batches, respectively. In China, a higher prevalence rate in sheep, reaching 96.9%, was reported (34). This relatively low prevalence rate is probably related to good management of pastures and the absence of co-grazing animals in the studied flock with other animals. Worms collected from the digestive tract of lambs were mainly represented by abomasum parasites. This is in agreement with the two studies conducted on sheep gastrointestinal parasites in North Tunisia (15, 16). We found herein that *Ostertagia sp*. was the predominant nematode genus (94.25%), unlike the two studies cited above, which reported a predominance of *Teladorsagia sp*. with an infection prevalence reaching 91.25 and 90.03%, respectively. This is probably related to the rainfall and ambient temperature, which constitute the two main factors conditioning the survival of the outdoor parasite stages in the soil (eggs and larvae).

## 5. Conclusion

We conclude that grazing on CONS A plots has no impact on sheep digestive parasitism compared to those grazing in CONV A. Similarly, we showed that there is almost no difference in lamb growth rate, carcass yield, or hematological parameters between lambs kept in the two pasture types. Further studies are needed to support these findings, especially on a larger animal sample, and to explore the impact of CONS A on other parasites (liver flukes and tick-borne infections) and other domestic animal herbivores. Unless these studies prove the opposite, sheep owners do not have to implement specific antiparasitic control measures on sheep grazing on CONS A.

## Data availability statement

The original contributions presented in the study are included in the article/supplementary material, further inquiries can be directed to the corresponding author.

## Ethics statement

The animal studies were approved by the approval code: CEEA-ENMV 65/22 approval date: December 29, 2022 Animal Ethics Committee – National School of Veterinary Medicine, AEC/IACUC, ENMV- Sidi Thabet, Tunisia. The studies were conducted in accordance with the local legislation and institutional requirements. Written informed consent was obtained from the owners for the participation of their animals in this study.

## Author contributions

Conceptualization: SE, MG, MR, and HC. Methodology and writing—review and editing: MG and SE. Validation: MG, MR, and HC. Formal analysis and investigation: SE, LS, and MD. Resources, writing—original draft preparation, and data curation: SE. Supervision and project administration: MG. Funding acquisition: MR. All authors have read and agreed to the published version of the manuscript.
